# Antibody Production and Immunoassay Development for Authenticating Chlorpheniramine Maleate Adulteration in Herbal Tea

**DOI:** 10.3390/foods13111609

**Published:** 2024-05-22

**Authors:** Jianhao Lin, Zhiwei Liu, Tian Guan, Yi Lei, Liangwen Pan, Xiaoqin Yu, Shiwei Zhang, Xin-An Huang, Hongtao Lei, Jiahong Chen

**Affiliations:** 1 Guangdong Provincial Key Laboratory of Food Quality and Safety/Nation-Local Joint Engineering Research Center for Machining and Safety of Livestock and Poultry Products, South China Agricultural University, Guangzhou 510642, China; 2 Guangdong Laboratory for Lingnan Modern Agriculture, Guangzhou 510642, China; 3 Guangzhou Institute of Food Inspection, Guangzhou 511410, China; 4 Animal, Plant and Food Inspection and Quarantine Technology Center, Shanghai Customs, Shanghai 200135, China; 5 Sichuan Institute of Food Inspection, Chengdu 610097, China; 6 National Nutrition Food Testing Center, Shenzhen Academy of Metrology and Quality Inspection, Shenzhen 518131, China; 7 Tropical Medicine Institute & South China Chinese Medicine Collaborative Innovation Center, Guangzhou University of Chinese Medicine, Guangzhou 510405, China; 8 Licheng Detection & Certification Group Co., Ltd., Zhongshan 528400, China

**Keywords:** chlorphenamine maleate, molecular simulation, adulteration, herbal teas

## Abstract

Chlorphenamine maleate is a prohibited additive found in herbal teas and health foods. Excessive intake of this substance can result in adverse health effects. In this study, two novel haptens, PEM and bepotastine (PB1), mimicking chlorphenamine maleate structure were designed and synthesized based on molecular simulation for developing two corresponding polyclonal antibodies (PEM-Ab and PB1-Ab), respectively. Afterward, an indirect competitive enzyme-linked immunosorbent assay (ic-ELISA) was developed to quickly and accurately detect chlorphenamine maleate in herbal teas using PB1-Ab, which has a high sensitivity and specificity. For chlorphenamine maleate, the half-maximal inhibitory concentration (IC_50_) and limit of detection (LOD) of PB1-Ab under ideal circumstances were found to be 1.18 µg/L and 0.07 µg/L, respectively. Besides, an environmentally friendly sample pre-treatment strategy was employed that allowed easy and effective elimination of complex matrices. The ic-ELISA method observed the average recovery rate from 87.7% to 94.0% with the variance coefficient (CV) ranging from 2.2% to 9.4%. Additionally, the identification of 25 commercially available herbal teas using liquid chromatography-tandem mass spectrometry (LC-MS/MS) further confirmed the validity of our detection. The results of the two methods are consistent. Overall, the proposed ic-ELISA could be an ultrasensitive and reliable method for chlorphenamine maleate adulterated in foods or exposure to the environment.

## 1. Introduction

Herbal tea, commonly used in Canton, Hong Kong, and Macao, is an herbal plant beverage with heat-clearing and detoxifying effects. It became one of the first batches of ethnic intangible cultural heritage in China in 2006 [1]. However, homemade herbal tea in bulk has often been found to be added synthetic drugs illegally in recent years to improve their efficacy, which greatly affects public health. In the current report of the illegal addition of herbal tea investigated by the National Market Supervision Administration of China, chlorphenamine maleate was the most frequently abused drug [2,3]. Chlorpheniramine maleate, known as a histamine Hl receptor antagonist, is widely used for the treatment of allergic reactions, hay fever, rhinitis, urticaria, asthma, etc., but excessive chlorpheniramine maleate exposure may come with serious adverse drug reactions, such as drowsiness, palpitation, skin ecchymosis, and increased bleeding tendency [4]. Therefore, it is necessary to monitor chlorpheniramine maleate adulteration in herbal tea.

Traditional chlorpheniramine maleate detection methods are generally based on chromatographic instrumental methods, including high-performance liquid chromatography (HPLC), gas chromatography (GC), and their mass spectrometry coupling techniques [5,6,7,8,9,10,11,12,13,14,15], which are highly dependent on large instruments and require multi-step sample pre-treatment and cannot meet the requirements of rapid field inspection. Immunoassays could be a good alternative method due to their rapidity, sensitivity, and cost-effectiveness advantages. Zhou et al. developed chlorpheniramine maleate monoclonal antibodies to prepare immunochromatographic test strips based on colloidal gold and fluorescent microsphere for chlorpheniramine maleate test in herbal tea; however, complicated operations and a relatively large amount of organic solvents were involved, which is time-consuming and not environmental-friendly [16]. Therefore, an on-site detection method endowed with facile and environmentally friendly pre-treatment operation is highly desired. In addition, high-quality antibody preparation is the key element for enzyme-linked immunosorbent assay (ELISA), which ultimately depends on rational hapten design. Nevertheless, there are no studies for the hapten design of chlorpheniramine maleate, and thus, how to obtain chlorpheniramine maleate antibodies with high sensitivity and specificity is also a key issue that needs to be solved urgently.

**Figure 1:**
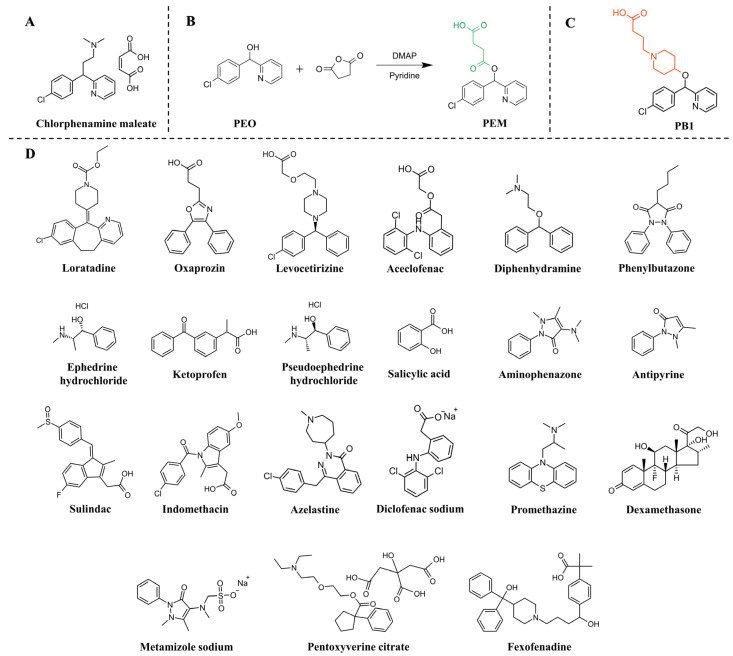
(**A**) The structure of chlorphenamine maleate. (**B**) Synthetic route of hapten PEM. (**C**) The structure of hapten PB1. (**D**) Chlorphenamine maleate and some structural and functional analogs.

In this study, two new haptens, PEM and bepotastine (PB1) were designed (Figure 1), and used molecular simulation techniques to analyze their main physical parameters, including dipole moment, electrostatic potential and so on. Polyclonal antibodies against two novel haptens were prepared, respectively. Through the comparative analysis of the structural characteristics of haptens and the performance evaluation of corresponding antibodies, the influence of haptens on the performance of antibodies was discussed. Furthermore, the antibodies were used to develop an indirect competitive ELISA (ic-ELISA) approach for detecting chlorphenamine maleate in herbal tea. The specificity and recovery results of the ic-ELISA method were verified by a standard LC-MS/MS method.

## 2. Materials and Methods

### 2.1. Reagents and Apparatus

The following compounds were obtained from Shanghai Bidepharm Co., Ltd. (Shanghai, China): Bepotastine (PB1), 4-Chlorophenyl-2-pyridinylmethanol (PEO), chlorphenamine maleate, diphenhydramine, diclofenac sodium, aceclofenac, dexamethasone, metamizole sodium, aminophenazone, and so on (Figure 1). Ovalbumin (OVA), bovine serum albumin (BSA), goat anti-rabbit IgG-horseradish peroxidase conjugate, 1-(3-dimethylaminopropyl)-3-ethylcarbodiimide hydrochloride (EDC) and N-hydroxysuccinimide (NHS) and isobutyl chloroformate were procured from Sigma-Aldrich Chemical Co. (St. Louis, MO, USA). The primary, secondary amine (PSA) sorbent and graphitized carbon black (GCB) sorbent were procured from Anpu Scientific Instrument Co., Ltd. (Shanghai, China). All other reagents were of analytical reagent grade or better and purchased from Macklin Biochemical Technology Co., Ltd. (Shanghai, China). The animal experiment was conducted in a laboratory that held a licence for the use of experimental animals and was conducted according to the principles of animal welfare (ethical approval number: 2019054, Figure S1).

### 2.2. Computational Chemistry Analysis of Haptens

All haptens energy minimum states were analyzed in GaussView 5.0 (Gaussian, Wallingford, CT, USA) [17]. The B3LYP density function and the 6-31G(d) basis set in Gaussian 09 (Gaussian, Wallingford, CT, USA) were used for optimization in the density-functional theory (DFT) calculations of haptens. The same software-based fundamental vibrations were subsequently employed to verify that the lowest energy conformations were indeed correct. Molecular descriptors, including hydrophobic constants (Log P) and molecular weights (MW), were extracted from haptens and chlorpheniramine maleate using ChemBio3D (PerkinElmer, Waltham, MA, USA). Molecular descriptors such as surface area (SA), polar surface area (PSA), and molecular polarity index (MPI) were extracted using Multiwfn 3.7 (dev) [18]. The electrostatic potential (ESP) mapped Van der Waals surface and solvent accessible surface area (SASA) are quantified by the VMD visualization program [19].

### 2.3. Preparation of Hapten and Artificial Antigens

The synthetic routes for hapten PEM were shown in Figure 1B, while hapten PB1 was obtained from commercial purchases (Figure 1C). The previously reported active ester method prepared most of the artificial antigens (PB1-KLH, PEM-OVA and PEM-BSA) in this study [20]. In addition, the coating antigen (PEO-OVA) was synthesized using the carbonyl diimidazole method; specific synthesis steps have also been described in previous reports [21]. Specific steps and identification of hapten are described in the Supplementary Material. Using UV-Vis spectral data, the final conjugate was validated.

### 2.4. Production of Chlorpheniramine Maleate Polyclonal Antibody

Polyclonal antibodies were prepared by immunizing New Zealand white rabbits. New Zealand white rabbits aged 3 months, one group consisted of a male and a female rabbit, each immunized with one kind of immunogen. Prior to the commencement of the experiment, blood was extracted from the ear veins of rabbits as a control. At the time of the initial immunization, 1 mg of immunogen (PEM-BSA or PB1-KLH) was dissolved in 1 mL of 0.01 mol/L PBS and emulsified with Freund’s complete adjuvant (1 mL). Subsequently, the emulsion was given by intradermal injection into the back of rabbits at the time of initial immunization. The Freund’s complete adjuvant was substituted with the same dose of the incomplete adjuvant in subsequent immunization. Following the fourth immunization, blood samples were collected from the ear veins of the rabbits. The collected blood was then centrifuged at 3000 rpm for 10 min. The supernatant was purified by octanoic acid-ammonium sulfate precipitation and stored at −20 °C until further use. The sensitivity of each antiserum was evaluated using ELISA by calculating antibody titer and inhibition rate. Antibody titer is defined as the dilution factor of the antiserum with the absorbance at 450 nm being situated at about 1.0–1.5 at a coating concentration of 1 μg/mL. The inhibition rate is defined as follows: inhibition rate = [1 − (B/B_0_)] × 100%. B_0_ was the mean absorbance of the wells in the absence of a competitor; B was the mean absorbance of the wells in the presence of a certain concentration of competitor.

### 2.5. Development and Optimization of ic-ELISA

To enhance the efficacy of the assay, a series of pivotal parameters influencing the ic-ELISA assay were meticulously calibrated, including the concentration of coating antigen and antibody, the buffer type, pH value, ionic strength, the reaction time of the primary antibody, and the dilution time of the goat anti-rabbit antibody [22]. The ELISA was developed using the regular procedure of ic-ELISA [22]. For further details, see the Supplementary Materials. The ic-ELISA calibration curve was developed as follows:

$$Y=\frac{\left(A-B\right)}{\left[1+{\left(\frac{X}{C}\right)}^{D}\right]}+B$$

The response at the high and low asymptotes of the curve, respectively, is designated as A and B. The concentration of the analyte that results in 50% inhibition is designated as C. The slope at the inflection point of the sigmoid is designated as D, and the calibration concentration is designated as X [23]. Calibration curves were generated using Origin Pro 8.5 (OriginLab Corp., Northampton, MA, USA). The limit of detection (LOD) of the method was defined as the concentration of the analyte that reduces the ODmax by 10% (IC_10_), with a linear range between IC_20_ and IC_80_ [24].

The specificity of the antibody was evaluated through cross-reactivity (CR) experiments. Several chlorpheniramine maleate structural analogs (Figure 1D) were selected for cross-reactivity (CR) testing by indirect competitive enzyme-linked immunosorbent assay (ic-ELISA). The CR were evaluated and calculated according to the following method:

$$CR(\%)=\frac{\left({IC}_{50}\mathrm{of}\mathrm{Chlorphenamine}\mathrm{maleate}\right)}{\left({IC}_{50}\mathrm{of}\mathrm{other}\mathrm{analogs}\right)}\times 100\%$$

### 2.6. Sample Preparation and Recovery

Combined with the previous method [25], the pre-treatment procedure of this study has been slightly modified, and the specific operations are as follows: 5 mL of uniform herbal tea sample was extracted into 10 mL centrifuge tube, then adsorbent (35 mg PSA and 65 mg GCB) was added and vortexed for 2 min to mix thoroughly. After standing, the supernatant was removed. For testing, the supernatant was passed through a 0.22 μm organic filtration membrane.

Herbal tea contains a large number of hydrophilic polysaccharides, tannins, proteins, and chlorophyll, with many components and complex pre-treatment procedures [25]. Samples of herbal tea that are bought at the neighborhood market are first identified by LC-MS/MS as “true negative” (Section 2.7). The samples of herbal tea were mixed with varying quantities of the standard solution of chlorpheniramine maleate. Subsequently, the sample pre-treatment technique was assessed by calculating the recovery rate and applying the sample preprocessing approach to eliminate the matrix interference impact of the sample.

### 2.7. Sample Adaptability and Confirmatory Test

The local herbal tea stores offered twenty-four distinct brands of bulk herbal tea, and all samples were pretreated using the above-described methods. Every sample was found using both the validated ic-ELISA and LC-MS/MS techniques. Triple (n = 3) determinations were made for each sample. The LC-MS/MS method’s protocol was adapted from State Standard, China BJS 201713 [26].

The sample preparation of LC-MS/MS is as follows: in a 50 mL centrifuge tube, 1 g of herbal tea (accurate to 0.0001 g) and 40 mL of chromatographic grade methanol were combined, vortexed for two minutes, and then ultrasonic extracted for thirty minutes. The mixture was run through a microporous filter membrane (0.22 μm, nylon membrane) once it had cooled to room temperature.

The confirmation test was performed using the LC-MS/MS. All the analyses were performed in triplicate. For details on the MS/MS parameters, see Table S1.

## 3. Results and Discussion

### 3.1. Hapten Design Strategy and Computational Validation

This study aimed to develop an antibody with high affinity and specificity for chlorpheniramine maleate. In the hapten design, the target analyte’s main structure should be retained to the maximum extent based on the principle of structural similarity [27]. Therefore, in this study, two original ring structures of chlorpheniramine maleate, including chlorobenzene ring and pyridine ring, were preserved for two new hapten structures. Herein, the conventional flexible linear spacer arm was designed to obtain hapten PEM, and the piperidine rings as spacer arms were introduced to hapten PB1 (Figure 1).

**Figure 2:**
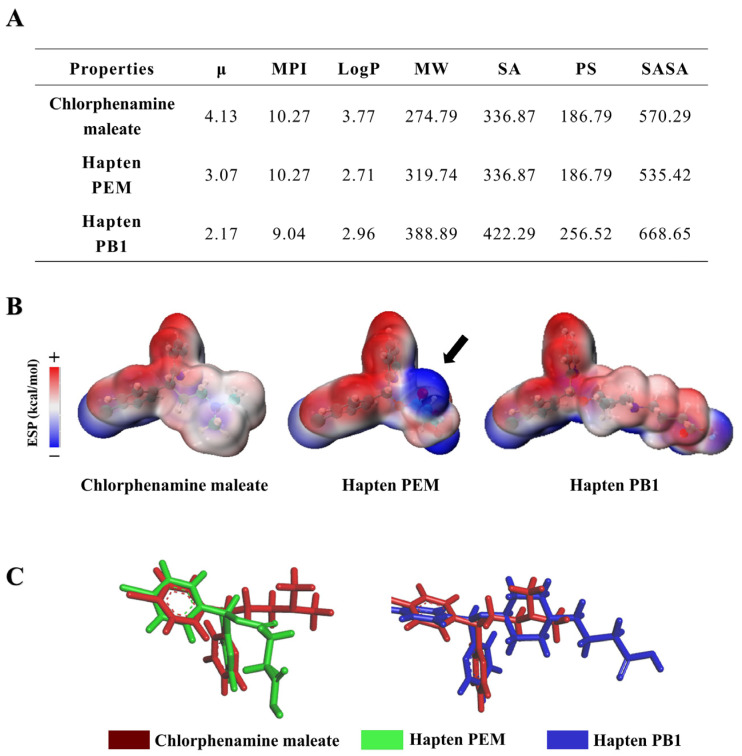
(**A**) Physicochemical parameters of chlorpheniramine maleate and haptens include μ, MPI, LogP, MW, SA, PSA, and SASA. (**B**) Surface electrostatic potential (ESP) of the chlorpheniramine maleate, haptens PEM, and hapten PB1. The difference in hapten PEM is pointed out by a black arrow. (**C**) Structural superposition of chlorpheniramine maleate (red), hapten PEM (green), and hapten PB1 (blue) at the lowest energy state.

To ensure the rationality and practicality of the designed hapten molecules, we initially employed computational chemistry to calculate their various physicochemical parameters (Figure 2A). By comparing the physicochemical parameters between two haptens, there are only subtle differences in μ, MPI, LogP, MW, SA, PS, and SASA. To better analyze the difference between the two hapten structures, the minimum energy conformation of the haptens was constructed to analyze the van der Waals surface electrostatic potential (ESP) distribution (Figure 2B). Based on the results of ESP distribution in the right part, where the difference in hapten PEM is pointed out by a black arrow, the position of the ESP on the epitope of hapten PEM changes significantly compared to the target chlorpheniramine maleate. In contrast, chlorpheniramine maleate and hapten PB1 shared a similar ESP distribution. When compared by computational chemistry software analysis, it was found that the electrostatic potential surface area distribution of hapten PEM under the energy-minimized structure did not have a high enough similarity to chlorpheniramine maleate (Figure 2B,C). The position of the spacer arm of hapten PEM lacks a nitrogen atomic group relative to chlorpheniramine maleate, which is a negative electron group in the electrostatic potential distribution analysis, while the spacer arm of hapten PEM is mainly a positive electron group. Based on the above analysis, we speculated that the antibodies induced after immunization with hapten PEM might have weak capability for the identification of chlorpheniramine maleate. In fact, this speculation was also verified after we synthesized hapten PEM and prepared the corresponding antibody (Table S2). As reported in previous studies, the introduction of spacer arms with spatial structure could improve the immunogenicity of immunogens [28]. Herein, the cyclic spacer arms with spatial structure were studied as follows. Compared with hapten with flexible linear spacer arm structure, the hapten PB1 introduced with piperidine rings as spacer arms was highly similar to the target compound chlorpheniramine maleate. So, spacer arms with piperidine rings were chosen to design hapten PB1 and the final hapten PB1-induced antibody with high specificity and sensitivity to chlorpheniramine maleate (Table S2). In conclusion, the computational chemistry data analysis theoretically supports our hypothesis that structural similarity affects the antibody’s performance. This new strategy for antigen design was further validated by antiserum performance evaluation.

### 3.2. Characterization of Antisera and Antibody against Chlorpheniramine Maleate

All artificial antigens PEM-BSA, PB1-KLH, PEO-OVA, and PEM-OVA were coupled by UV-vis identification (Figure S2), and the 280 nm maximum peak of the contrast analyzed conjugates changed significantly, indicating that the hapten was successfully coupled to the carrier protein. Rabbit antisera were collected and evaluated for titer and inhibition in ic-ELISA against homologous and heterologous coating antigens following animal vaccination with artificial antigens. As demonstrated in Table S2, the highest inhibition was observed when PB1-KLH was used. It has been demonstrated that the inhibition rate of hapten PB1 is superior to that of hapten PEM. This may be attributed to the spacer arm of PB1 comprising a piperazine ring structure and the influence of nitrogen atomic groups, as evidenced by the computational chemistry analysis. The results of the antiserum screening indicated that the hapten PEM and PB1 had long-linked arms compared to the original chlorpheniramine maleate structure. The antibodies obtained were capable of recognizing chlorpheniramine maleate, exhibiting higher antibody titers. Nevertheless, the presence of a nitrogen atom on the chlorpheniramine maleate structure may also influence its antigen-antibody recognition efficacy. However, the absence of nitrogen atomic groups in the PEM structure resulted in the poor performance of its antibody due to the structural differences between the antibody and the coating antigens. These differences manifested in a low rate of antibody inhibition as a result of the key interactions between the antibody and the coating antigens, such as hydrogen bond interactions, hydrophilic interactions, and electrostatic interactions. In contrast, the link arm of PB1 contains a nitrogen atomic polar group and has a spatial structure that facilitates the recognition of antibodies induced by the antigen. Consequently, the affinity between the antibody and the two heterogeneous envelope antigens was diminished, while the competition of chlorpheniramine maleate was relatively enhanced [29]. In addition, for the PB1-KLH antibody, the inhibition rate of the heterogenous combination using PEO-OVA is better than that of the combination using PEM-OVA, indicating that the structural difference may contribute to immunoassay sensitivity. Overall, the results of both studies suggest that retaining part of the maternal nuclear structure of the drug and linking circular arms with polar groups could serve as a viable antigen design strategy to improve chlorpheniramine maleate inhibition.

### 3.3. Optimization and Development of ic-ELISA

**Figure 3:**
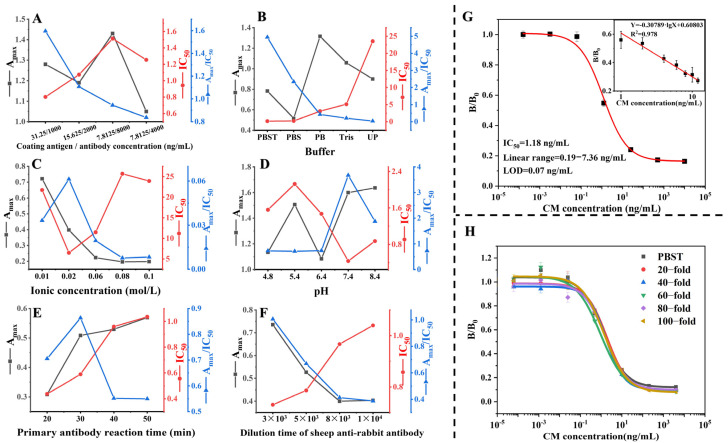
The optimization of the physicochemical parameters and sample pre-treatment method on the ic-ELISA. Optimization of (**A**) the coating antigen/antibody concentration (µg/L), (**B**) the selection of the buffer, (**C**) ionic strength, (**D**) pH value, (**E**) primary antibody reaction time, and (**F**) dilution of goat anti-rabbit antibody. (**G**) The standard curve of chlorpheniramine maleate was assessed by ic-ELISA in an optimized buffer (n = 3). (**H**) matrix effect of Guangdong herbal tea samples on ic-ELISA performance (n = 3). PBS, Phosphate-buffered saline. PBST, PBS with 0.05% Tween-20. Up, Ultrapure water. Tris, Tris-HCL buffer.

The efficiency of ic-ELISA may be influenced by the physicochemical factors associated with the buffer. In order to optimize the performance of the ic-ELISA, a number of key factors were selected and assessed, including the concentration of coating antigen and antibody, the buffer type, pH value, ionic concentration, the reaction time of the primary antibody, and the dilution time of the goat anti-rabbit antibody. The impact of these variables was quantified by measuring the maximum signal (Amax) and IC_50_ of the standard curve under varying conditions. In this study, the Amax/IC_50_ ratio was employed as the principal criterion for evaluating the performance of the ic-ELISA. A higher Amax/IC_50_ ratio was found to be indicative of enhanced performance [27]. Following the optimization of these factors, the highest sensitivity was observed when the coating antigen/antibody concentration was 3.125 µg/L and 1000 µg/L, respectively, using 0.02 mol/L PBST at pH = 7.4. The optimum conditions for goat anti-rabbit antibody’s primary antibody reaction time and dilution time are 30 min and 3000 fold, respectively (Figure 3A–F). The IC_50_ and linear range of the established ic-ELISA for chlorpheniramine maleate were 1.18 µg/L and 0.19–7.36 µg/L, and the LOD was 0.07 µg/L (Figure 3G).

The specificity of ic-ELISA was determined by analysis of chlorpheniramine maleate and other analogs, and the cross-reactivity (CR) of the associated compounds was calculated under ideal working conditions (Table 1). All related compounds showed low CR even at high levels (2000 µg/L), indicating that the developed ELISA showed good specificity for chlorpheniramine maleate.

### 3.4. Recovery Analysis

Following the pre-treatment phase, the samples were diluted to four levels (20, 40, 60, 80, 100-fold) with an optimized PBST buffer for matrix effect testing. As shown in Figure 3H. The effects of matrix effects can be essentially removed at a 100-fold dilution.

Three levels of chlorpheniramine maleate (2, 4, and 6 µg/L) were added to the sample for ic-ELISA validation. All added samples were analyzed using ic-ELISA and LC-MS/MS; the results are summarized in Table 2. The ic-ELISA method observed the average recovery rate from 87.7% to 94.0%, with the variance coefficient (CV) ranging from 2.2% to 9.4%. The average recovery rate of the LC-MS/MS method was 106.8–112.4%, while the average coefficient of variable (CV) was 0.1–0.7%. The results showed that the accuracy of the ic-ELISA method was comparable to the LC-MS/MS method.

### 3.5. Real Sample Analysis

This study also sampled 25 commercial bulk herbal tea varieties in Guangzhou and compared them by the established ic-ELISA method and the LC-MS/MS method recommended by the China National Supplementary Inspection method (Table 3). For all the tested samples, chlorpheniramine maleate was found in four samples, one of which had a high chlorpheniramine maleate content of 5.02 mg/kg, and more than 500 times above the legal standard, indicating that there are still risks in the bulk herbal tea market and supervision needs to be strengthened. The ic-ELISA approach developed in this work may be employed as a quantitative detection method to find the unlawfully added chlorpheniramine maleate in bulk Chinese herbal tea since the detection findings of the two methods are also consistent.

## 4. Conclusions

In this study, we successfully designed and evaluated two new chlorpheniramine maleate haptens and described the hapten structures’ main physical parameters, including dipole moment, electrostatic potential, SA, PA, and SASA, by molecular simulation techniques. The combined results of the antibody performance evaluation and the analysis indicated that increasing the hapten’s molecular volume and structural similarity by spacer arms may be a promising new strategy. This strategy was finally demonstrated by computational chemistry and antibody performance evaluation. A simple, user-friendly ELISA method was developed and employed to screen a range of commercially available bulk herbal teas for the presence of illegally added chlorpheniramine maleate. This was achieved by utilizing an antibody prepared from the hapten PB1. Following a simple pre-treatment step, the method may be employed for the rapid and sensitive detection of chlorpheniramine maleate in herbal teas. The LOD is 0.07 µg/L. The recovery of the samples ranged from 87.7% to 94.0%. The results of the ic-ELISA were validated by standard LC-MS/MS, which demonstrated that the method was both accurate and reproducible. The technique is ideal for the high-throughput, ultrasensitive, low-cost screening of real samples.

## Figures and Tables

**Table 1:** Specificity evaluation of antibody.

Analyte	IC_50_ (µg/L)	CR (%)
Chlorpheniramine maleate	1.18	100.00
Loratadine	>2000	<0.01
Oxaprozin	>2000	<0.01
Levocetirizine	>2000	<0.01
Aceclofenac	>2000	<0.01
Diphenhydramine	>2000	<0.01
Phenylbutazone	>2000	<0.01
Ephedrine hydrochloride	>2000	<0.01
Ketoprofen	>2000	<0.01
Pseudoephedrine hydrochloride	>2000	<0.01
Salicylic acid	>2000	<0.01
Aminophenazone	>2000	<0.01
Antipyrine	>2000	<0.01
Sulindac	>2000	<0.01
Indomethacin	>2000	<0.01
Azelastine	>2000	<0.01
Diclofenac sodium	>2000	<0.01
Promethazine	>2000	<0.01
Dexamethasone	>2000	<0.01
Metamizole sodium	>2000	<0.01
Pentoxyverine citrate	>2000	<0.01
Fexofenadine	>2000	<0.01

**Table 2:** Recoveries of chlorpheniramine maleate from spiked herbal tea samples by ic-ELISA (n = 3) and LC-MS/MS ^a^.

Samples No.	Spiked Levels (µg/L)	ic-ELISA			LC-MS/MS		
		Measured (µg/L) (Mean ± SD ^a^)	Recovery ^c^ (%)	CV ^b^ (%)	Measured (µg/L)	Recovery (%)	CV (%)
Blank	0	ND ^c^	NC ^d^	NC	ND	NC	NC
1	2	1.88 ± 0.07	94.0	3.8	2.25 ± 0.02	112.4	0.7
2	4	3.51 ± 0.08	87.7	2.2	4.31 ± 0.02	107.8	0.3
3	6	6.59 ± 0.62	109.9	9.4	6.41 ± 0.01	106.8	0.1

^a^ SD, standard deviation. ^b^ CV, coefficient of variance. ^c^ ND, not detected. ^d^ NC, not calculated.

**Table 3:** Recoveries of chlorpheniramine maleate from real herbal tea samples by ic-ELISA (n = 3) and LC-MS/MS.

Samples No.	Spiked Levels (µg/L)	ic-ELISA			LC-MS/MS		
		Measured (μg/kg) (Mean ± SD ^a^)	Recovery (%)	CV ^b^ (%)	Measured (µg/L)	Recovery (%)	CV (%)
1	0	ND ^c^	NC ^d^	NC	ND	NC	NC
2	0	ND	NC	NC	ND	NC	NC
3	0	ND	NC	NC	ND	NC	NC
4	0	ND	NC	NC	ND	NC	NC
5	0	ND	NC	NC	ND	NC	NC
6	0	ND	NC	NC	ND	NC	NC
7	0	ND	NC	NC	ND	NC	NC
8	0	128.59 ± 2.38	NC	1.85	130.00	NC	NC
9	0	111.88 ± 6.09	NC	5.44	120.00	NC	NC
10	0	ND	NC	NC	ND	NC	NC
11	0	ND	NC	NC	ND	NC	NC
12	0	ND	NC	NC	ND	NC	NC
13	0	ND	NC	NC	ND	NC	NC
14	0	ND	NC	NC	ND	NC	NC
15	0	ND	NC	NC	ND	NC	NC
16	0	ND	NC	NC	ND	NC	NC
17	0	ND	NC	NC	ND	NC	NC
18	0	ND	NC	NC	ND	NC	NC
19	0	ND	NC	NC	ND	NC	NC
20	0	ND	NC	NC	ND	NC	NC
21	0	5373 ± 24	NC	0.44	5020	NC	NC
22	0	110.40 ± 3.56	NC	3.22	110.00	NC	NC
23	0	ND	NC	NC	ND	NC	NC
24	0	ND	NC	NC	ND	NC	NC
25	0	ND	NC	NC	ND	NC	NC

^a^ SD, standard deviation. ^b^ CV, coefficient of variance. ^c^ ND, not detected. ^d^ NC, not calculated.

## Data Availability

The original contributions presented in the study are included in the article/Supplementary Material, further inquiries can be directed to the corresponding authors.

## Supplementary Materials

The following supporting information can be downloaded at: https://www.mdpi.com/article/10.3390/foods13111609/s1, Figure S1: Results of ethical review of animal experiments; Figure S2: The result of UV-Vis spectroscopy of (A) PB1, KLH, and PB1-KLH; (B) PEO, OVA, and PEO-OVA; (C) PEM, BSA, and PEM-BSA; and (D) PEM, OVA, and PEM-OVA; Figure S3: Liquid chromatography and calibration curve of chlorpheniramine maleate (CM); Table S1: MS/MS Conditions for chlorpheniramine maleate; Table S2: Characterization of rabbit antisera with homologous and heterologous coating antigens for selection of the best antibody.

## Abbreviations

ELISA: enzyme-linked immunosorbent assay, IC_50_: half inhibitory concentration, LC-MS/MS: liquid chromatography with tandem mass spectrometry, CR: cross-reactivity, SD: standard deviation, CV: the coefficient of variable, μ: dipole moment, MPI: molecular polarity index, MW: molecular weight, E: energy, SA: surface area, PSA: polar surface area, SASA: solvent accessible surface areas, LOD: The limit of detection.
