# Supplementary material for: Antibody Production and Immunoassay Development for Authenticating Chlorpheniramine Maleate Adulteration in Herbal Tea
Source: Foods. 2024 May 22;13(11):1609. doi: 10.3390/foods13111609 (PMC11172221; doi:10.3390/foods13111609)
Supplement: Supplementary file 1 [file foods-13-01609-s001.zip › foods-2992721-supplementary.pdf]

Supplementary Materials

Antibody Production and Immunoassay Development for Authenticating Chlorpheniramine Maleate Adulteration in Herbal Tea

Jianhao Lin [1,2], Zhiwei Liu [1,2], Tian Guan [1,2], Yi Lei [3], Liangwen Pan [4], Xiaoqin Yu [5], Shiwei Zhang [6], Xin-An Huang [7], Hongtao Lei [1,2,8,*] and Jiahong Chen [1,2,*]

[1] Guangdong Provincial Key Laboratory of Food Quality and Safety/Nation-Local Joint Engineering Research Center for Machining and Safety of Livestock and Poultry Products, South China Agricultural University, Guangzhou 510642, China; 13726640373@163.com (J.L.); liuzhiwei2021888@163.com (Z.L.); guantian@scau.edu.cn (T.G.)
[2] Guangdong Laboratory for Lingnan Modern Agriculture, Guangzhou 510642, China
[3] Guangzhou Institute of Food Inspection, Guangzhou 511410, China; leiy04@foxmail.com
[4] Animal, Plant and Food Inspection and Quarantine Technology Center, Shanghai Customs, Shanghai 200135, China; panlw888@126.com
[5] Sichuan Institute of Food Inspection, Chengdu 610097, China; 113343838@foxmail.com
[6] National Nutrition Food Testing Center, Shenzhen Academy of Metrology and Quality Inspection, Shenzhen 518131, China; zsw_8506@163.com
[7] Tropical Medicine Institute & South China Chinese Medicine Collaborative Innovation Center, Guangzhou University of Chinese Medicine, Guangzhou 510405, China; xinanhuang@gzucm.edu.cn
[8] Licheng Detection & Certification Group Co., Ltd., Zhongshan 528400, China
* Correspondence: hongtao@scau.edu.cn (H.L.); jiahongchen@scau.edu.cn (J.C.); Tel.: +86-20-8528-3925 (H.L.); +86-20-8528-0266 (J.C.); Fax: +86-20-8528-0270 (H.L. & J.C.)

2.1 Reagents and Apparatus

In this work, the following solutions and buffers were employed: (1) 0.01 M Phosphate-buffered saline (PBS) with a pH of 7.4; (2) 0.01 M carbonate buffer (CB) with a pH of 9.6; (3) PBST (0.01 M PBS with 0.05% Tween-20, pH 7.4); (4) blocking buffer to be made with 0.1% hydrolyzed protein (w/v) and 0.01 M PBST; and (4) 10% H_2SO_4 (used as the stop reagent). Purified water was prepared using a Milli-Q water purification system (Millipore, Bedford, MA). The concentration of antibody was detected using a NanoDrop 2000c spectrophotometer (Thermo Fisher, Shanghai, China). Absorbance was measured at a wavelength of 450 nm using a Multiskan MK3 microplate reader (Thermo Fisher, Shanghai, China). The chromatography was manipulated on the LC-MS/MS system including a Shimadzu LC- 30 system (Kyoto, Japan), AB QTRAP4500 triple quadrupole mass spectrometer (AB SCIEX, USA), and a Phenomenex Luna Omega chromatographic column (C18, 2.1×100 mm, 1.6 μm). Nuclearmagnetic resonance (NMR) spectra were recorded with either a DRX-400 or DRX-600 NMR spectrometer (Bruker, Rheinstetten, Germany).

2.2 Preparation of hapten and artificial antigens

PEM was synthesize as follows: PEO (200 mg, 0.91 mmol), Succinic anhydride (455 mg, 4.55mmol), and 4-dimethylamino pyridine (11 mg, 90 μmol) were dissolved in anhydrous pyridine (1 mL), then stirred at room temperature for 24 h. Saturated sodium carbonate (10 mL) was added to the mixture, which was stirred for a further 10 min. The mixture was then extracted with ethyl acetate for 3 times, and the aqueous layer was cooled and acidified to pH 3.0 with HCl (2 mol/L). After filtration, the collected precipitate was washed to neutrality using saturated

saline, the desired target was obtained under vacuum-dried, named PEM. The structure of hapten PEM was confirmed by MS and nuclear magnetic resonance (NMR) analyses. The following results were obtained from the ESI-MS analysis (positive): m/z 320.1 1H NMR (600 MHz, Methanol-d4) δ 8.09 – 8.05 (m, 8H), 7.40 (d, J = 8.4 Hz, 1H), 7.34 – 7.27 (m, 2H), 6.71 – 6.66 (m, 8H), 3.07 (s, 24H), 2.64 – 2.56 (m, 1H), 1.34 (s, 2H), 1.30 (t, J = 4.2 Hz, 6H), 0.94 – 0.86 (m, 3H).

Active ester method: Briefly, the hapten PB1 (50 μmol) was reacted with N-hydroxysuccinimide (50 μmol) in presence of N, N, -dicyclohexylcarbodiimide (50 μmol) in anhydrous N, N-dimethylformamide (2 mL). After overnight incubation at room temperature in the dark, the mixture was incubated for another 6 h with KLH, BSA or OVA (75 mg) previously dissolved in 5 mL of borate buffer (0.1 M, pH 9.0). Finally, the solutions were dialyzed against 0.01 mol/L PBS (pH 7.4) over 3 days at 4°C with three changes each day, and finally stored at -20°C until use. The protein conjugates were identified by an ultraviolet spectrophotometer, and UV-vis spectral data was used to confirm the structure of the final conjugates.

Carbonyl diimidazole method:Briefly, the PEO (50 μmol) was reacted with N, N-base diimidazole (200 μmol) in anhydrous N, N-dimethylformamide (0.3 mL). After 24 h incubation at room temperature in the dark, the mixture was incubated for another 6 h with OVA (10 mg) previously dissolved in 0.8 mL of carbonate buffer (0.1 mol/L, pH 9.0). Finally, the solutions were dialyzed against 0.01 mol/L PBS (pH 7.4) over 3 days at 4°C with three changes each day. The protein conjugates were then characterized by UV-Vis, which was finally stored at -20 ℃ until use.

2.3 ELISA Protocol

The coating antigens diluted in carbonate buffer were added into microplates for 100 μL/well and incubated at 37 °C for 12 h. After the microplates were washed twice, the blocking solution for 200 μL/well was added to block the unbound active sites at 37 °C for 3 h, and dried at 37 °C for 1 h. 50 μL of target analyte standards or diluted sample solutions and 50 μL of diluted antibodies in PBST were added into each well and incubated at 37 °C for 40 min. and then the wells were washed five times. 100 μL of HRP labeled secondary antibody (diluted 5000 times in PBST) was then added to the each well and incubated at 37 °C for 40 min. Before the addition of 100 μL of TMB-based substrate solution, the wells were washed again for five times. The enzymatic chromogenic reaction proceeds for 10 min and then stopped by adding 50 μL of 2 M H_2SO_4. Finally, absorbance at 450 nm was recorded.

2.4 Sample adaptability and confirmatory test

Phenomenex Luna Omega C18 chromatographic column (100 mm × 2.1 mm, 1.6 μm) was used to resolve the analyte. The column temperature was set to 40℃. The mobile phase was consisted of aqueous solution containing 0.1% (v/v) formic acid and 100% acetonitrile solution. All mobile phases were sonicated for 5 min before use. Equal elution (80% formic acid + 20% acetonitrile solution, volume ratio), flow phase flow rate was set to 0.3 mL/min and sample injection was 2 μL.

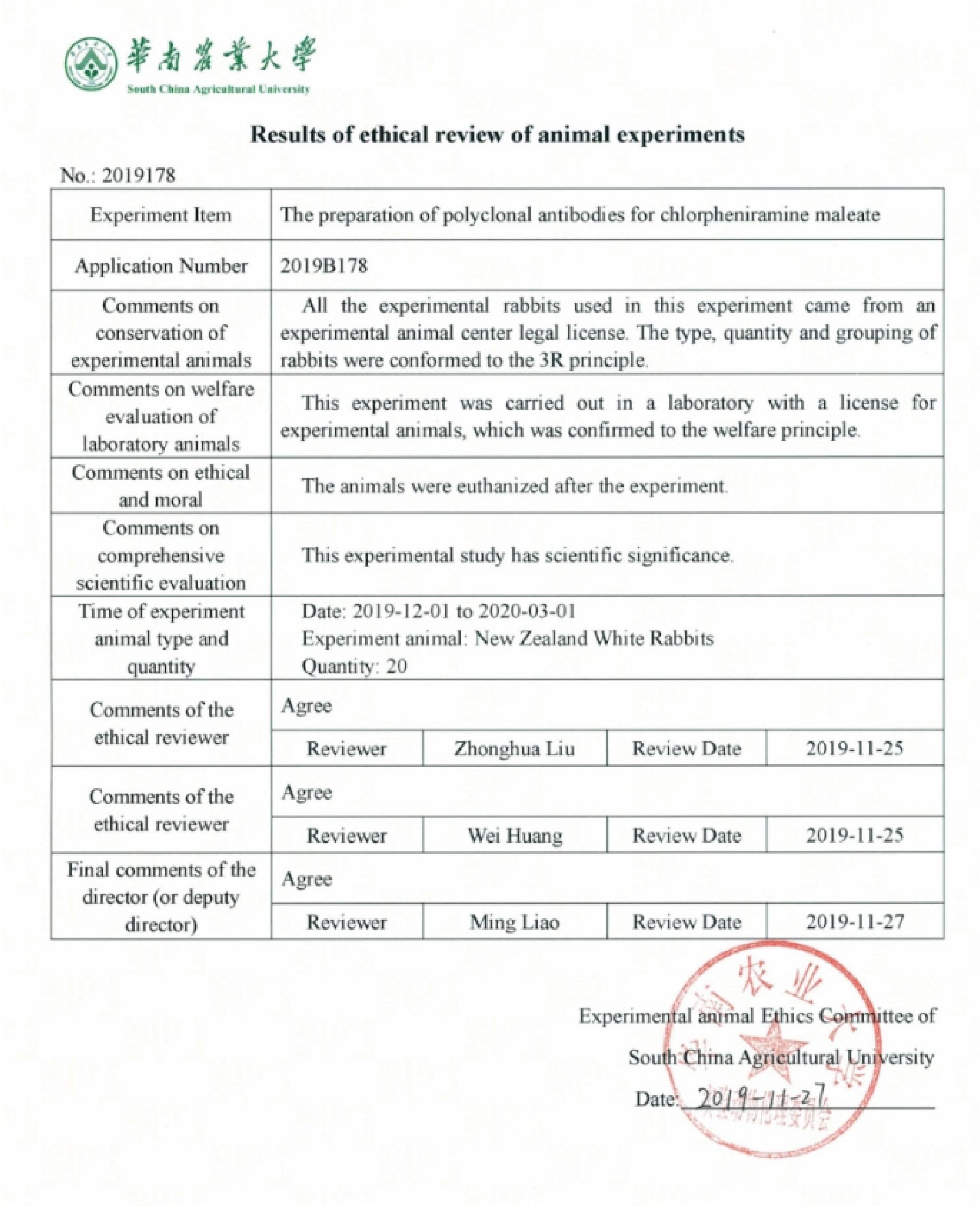

华南农业大学

South China Agricultural University

Results of ethical review of animal experiments

No.: 2019178

Experiment Item	The preparation of polyclonal antibodies for chlorpheniramine maleate			
Application Number	2019B178			
Comments on conservation of experimental animals	All the experimental rabbits used in this experiment came from an experimental animal center legal license. The type, quantity and grouping of rabbits were conformed to the 3R principle.			
Comments on welfare evaluation of laboratory animals	This experiment was carried out in a laboratory with a license for experimental animals, which was confirmed to the welfare principle.			
Comments on ethical and moral	The animals were euthanized after the experiment.			
Comments on comprehensive scientific evaluation	This experimental study has scientific significance.			
Time of experiment animal type and quantity	Date: 2019-12-01 to 2020-03-01<br>Experiment animal: New Zealand White Rabbits<br>Quantity: 20			
Comments of the ethical reviewer	Agree			
	Reviewer	Zhonghua Liu	Review Date	2019-11-25
Comments of the ethical reviewer	Agree			
	Reviewer	Wei Huang	Review Date	2019-11-25
Final comments of the director (or deputy director)	Agree			
	Reviewer	Ming Liao	Review Date	2019-11-27

Experimental animal Ethics Committee of
South China Agricultural University
Date: 2019-11-27

Figure S1 Results of ethical review of animal experiments.

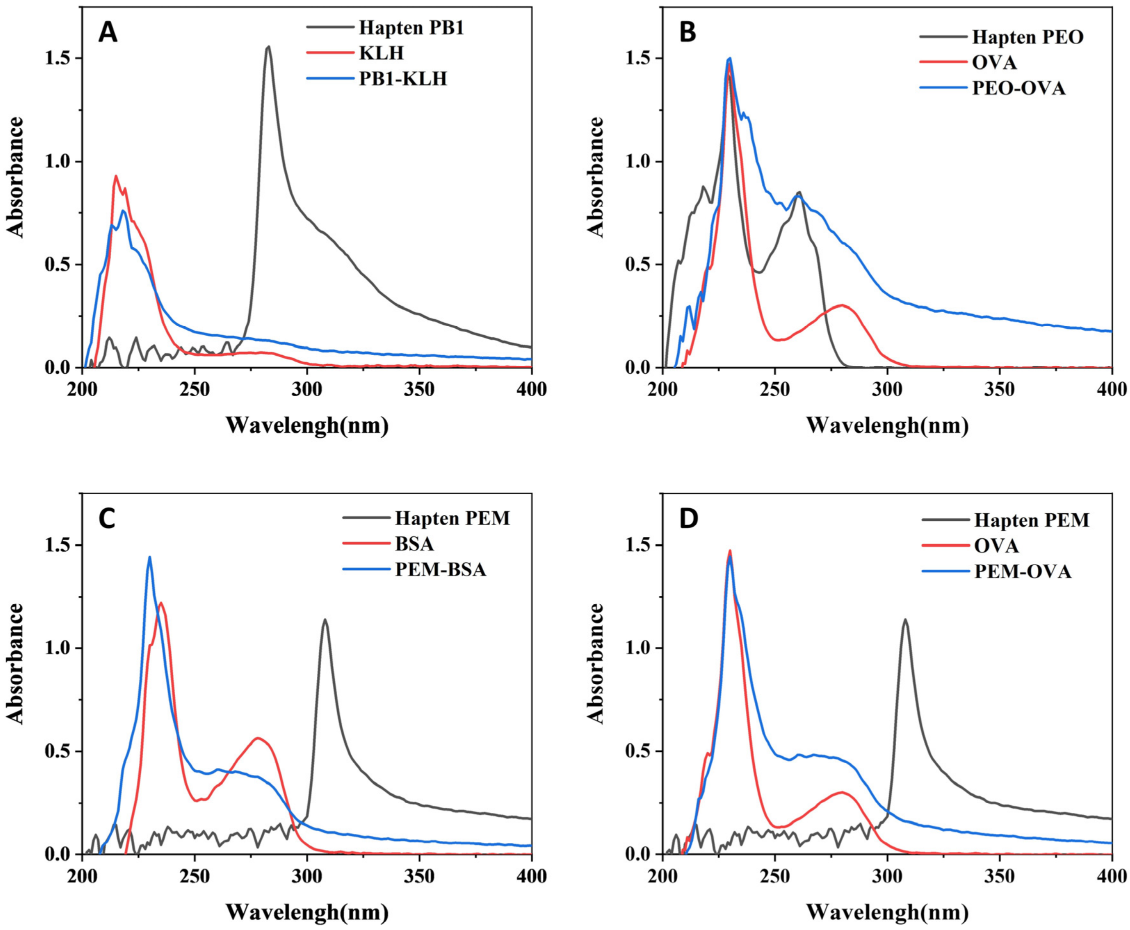

Figure S2 The result of UV-Vis spectroscopy of (A) PB1, KLH, and PB1-KLH; (B) PEO, OVA, and PEO-OVA; (C) PEM, BSA, and PEM-BSA; and (D) PEM, OVA, and PEM-OVA.

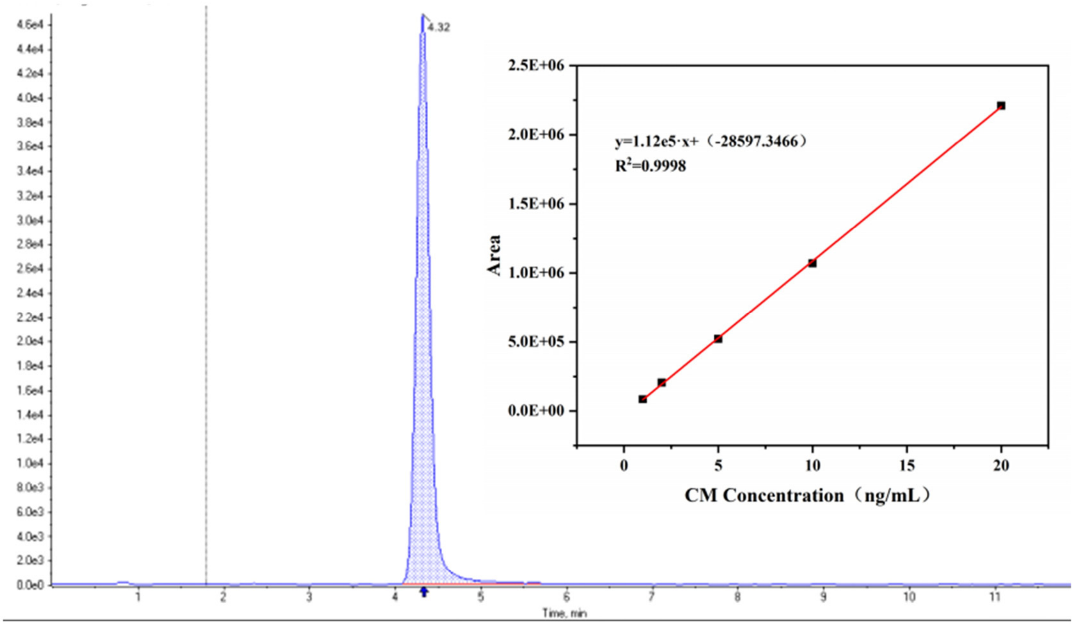

Figure S3 Liquid chromatography and calibration curve of chlorphenamine maleate (CM).

Table S1 MS/MS conditions for chlorphenamine maleate.

Analyte	RT/min	Precursor ion /(m/z)	Product ion /(m/z)	DP / eV	CE / V
chlorphenamine maleate	4.31	275*	230.0	62.00	23.00
		275	167.1	44.00	53.00

*Quantitative ion

Table S2 Characterization of rabbit antisera with homologous and heterologous coating antigens for selection of the best antibody.

Immunogen	PEM-BSA		PB1-KLH	
Coating antigen	Titer[a]（×10^3）	Inhibition[b]（%）	Titer（×10^3）	Inhibition（%）
PEM-OVA	256	43.32	512	75.66
PEO-OVA	<1	ND[c]	1024	93.72

[a] Titer is defined as dilution factor of antiserum with the absorbance at 450 nm being situated at about 1.0-1.5 at coating concentration of 1000 ng/mL.

[b] Percentage inhibition was expressed as follow: inhibition (%) = $[1-(B/B_0)] \times 100$. B_0 was mean absorbance of the wells in the absence of competitor; B was mean absorbance of the wells in the presence of certain concentration of competitor.

[c] ND, no detected.
